# Potential of Andean Grains as Substitutes for Animal Proteins in Vegetarian and Vegan Diets: A Nutritional and Functional Analysis

**DOI:** 10.3390/foods14172987

**Published:** 2025-08-27

**Authors:** Jhonsson Luis Quevedo-Olaya, Marcio Schmiele, María Jimena Correa

**Affiliations:** 1 Programa de Doctorado en Ingeniería Agroindustrial, Mención Transformación Avanzada de Granos y Tubérculos Andinos, Universidad Nacional del Santa, Nuevo Chimbote 02712, Peru; 2025818006@uns.edu.pe; 2 Institute of Science and Technology, Federal University of Jequitinhonha and Mucuri Valleys, Diamantina 39100-000, Brazil; marcio.sc@ict.ufvjm.edu.br; 3 School of Food Engineering, University of Campinas, Campinas 13083-887, Brazil; 4 Centro de Investigación y Desarrollo en Ciencia y Tecnología de los Alimentos (CIDCA), Facultad de Ciencias Exactas, Universidad Nacional de La Plata, CIC, CONICET, 47 y 116, La Plata 1900, Argentina

**Keywords:** Andean pseudocereals, kañiwa, amaranth, quinoa, plant protein, amino acid profile, processing technologies, functional foods, protein substitution

## Abstract

The growing demand for sustainable protein sources has boosted interest in Andean pseudocereals, particularly quinoa (*Chenopodium quinoa*), cañihua (*Chenopodium pallidicaule*), and kiwicha (*Amaranthus caudatus*), due to their complete nutritional profile, high digestibility, and low allergenic potential. Their inclusion in vegetarian and vegan diets represents a viable alternative that can replace animal proteins without compromising on nutritional quality. This study presents a critical review of indexed scientific literature analyzing essential amino acid composition, protein quality values—such as PDCAAS (Protein Digestibility-Corrected Amino Acid Score) and DIAAS (Digestible Indispensable Amino Acid Score)—and the impact of various processing technologies on the functionality of Andean proteins. Results show that these grains contain between 13 and 18 g of protein per 100 g of dry product and provide adequate levels of lysine, methionine, and threonine, meeting FAO (Food and Agriculture Organization) requirements for adult nutrition. Processes such as germination, fermentation, enzymatic hydrolysis, and extrusion have demonstrated improvements in both amino acid bioavailability and functional properties of proteins, enabling their application in gluten-free breads, meat analogs, and functional beverages. Furthermore, emerging strategies such as nanotechnology, bioactive peptide generation, and gene editing via CRISPR (Clustered Regularly Interspaced Short Palindromic Repeats)—a precise genome editing tool—open new possibilities for enhancing the nutritional and functional value of pseudocereals in the food industry. Taken together, the findings consolidate the strategic role of Andean grains as key ingredients in the development of sustainable, functional, and plant-based foods.

## 1. Introduction

The current food system faces unprecedented pressures related to climate change, population growth, and the need to transition towards more sustainable and resilient production models. In this context, interest has intensified in developing plant-based food systems aimed at reducing the environmental impact of livestock farming, improving the efficiency of natural resource use, and offering nutritionally adequate alternatives to animal proteins [1,2,3].

However, one of the main challenges of these strategies lies in adequately meeting protein requirements, especially in vegetarian or vegan diets. Plant-based proteins often lack one or more essential amino acids, show low digestibility, or have limited techno-functional properties that hinder their use in complex industrial applications [4,5,6]. These limitations have driven the search for alternative ingredients with improved nutritional and functional profiles, capable of partially or fully replacing animal proteins in various food matrices.

Within this emerging landscape, Andean grains—particularly quinoa (*Chenopodium quinoa*), cañihua (*Chenopodium pallidicaule*), and kiwicha (*Amaranthus caudatus*)—stand out not only for their exceptional composition but also for their adaptability to extreme agroclimatic conditions and their sociocultural value in high Andean regions [7,8,9]. These pseudocereals have higher protein content than many conventional cereals, with amino acid profiles comparable to casein, and are rich in minerals, dietary fiber, and bioactive compounds [9,10,11].

They have also demonstrated outstanding functional properties, such as solubility, emulsification, gelation, and foaming capacity, making them attractive for the development of innovative products like meat analogs, plant-based milks, pastas, and functional bakery goods [12,13,14]. Processing techniques such as germination, extrusion, fermentation, and chemical modification have been employed to enhance their functionality and reduce antinutritional factors, thereby broadening their technological viability [15,16,17].

Their cultivation also contributes to agrobiodiversity and environmental sustainability, due to low water requirements, tolerance to poor soils and high altitudes, and integration into traditional agricultural systems [18]. From an economic and social perspective, they represent an opportunity to strengthen local value chains and foster a circular bioeconomy in highland communities [2].

Therefore, this review aims to evaluate the comprehensive potential of quinoa, cañihua, and kiwicha as functional and nutritional substitutes for animal proteins, covering (i) protein composition and amino acid profile, (ii) digestibility and bioavailability, (iii) techno-functional properties, (iv) processing technologies, (v) food applications, (vi) environmental impact, and (vii) socioeconomic considerations. The analysis is based on more than 80 scientific publications, prioritizing experimental studies, systematic reviews, and recent comparative studies from the last 10 years.

## 2. Nutritional and Protein Profile of Andean Grains

### 2.1. Crude Protein Content and Essential Amino Acid Composition

Andean pseudocereals such as quinoa (*Chenopodium quinoa*), cañihua (*Chenopodium pallidicaule*), and kiwicha (*Amaranthus caudatus*) are characterized by a crude protein content ranging from 15.0 to 16.3% on a dry basis, surpassing traditional cereals such as rice (7.0%) and maize (9.0%) [9,19,20]. In addition, they exhibit a balanced essential amino acid profile, with lysine, methionine, and threonine levels that meet or exceed FAO requirements for high-quality nutritional proteins [21].

When compared with other protein sources, pseudocereals present higher lysine content than wheat (260 mg/100 g) and rice (300 mg/100 g), although lower than egg (930 mg/100 g) and beef (880 mg/100 g) [22,23]. Milk, despite having a lower total protein content (3.3% d.b.), maintains a highly digestible and high biological value amino acid profile [21,24].

Including high-Andean legumes and oilseeds such as tarwi (*Lupinus mutabilis*), pajuro (*Erythrina edulis*), and sacha inchi (*Plukenetia volubilis*) in the comparison highlights the potential of these species to diversify and complement the essential amino acid contribution in plant-based diets. Tarwi stands out for its high protein content (42% d.b.) and its lysine- and leucine-rich profile [25]. Pajuro shows intermediate protein values (21.5% d.b.) and a balanced amino acid composition [26], while sacha inchi, with 27% protein, combines a good-quality protein profile with a significant supply of polyunsaturated fatty acids [27,28].

Table 1 summarizes the approximate crude protein content and essential amino acid composition of these sources, expressed in mg/100 g dry product, evidencing the competitiveness of Andean pseudocereals compared to other plant and animal proteins.

### 2.2. Protein Digestibility and Bioavailability

PDCAAS (Protein Digestibility-Corrected Amino Acid Score) and DIAAS (Digestible Indispensable Amino Acid Score) are tools used to evaluate protein nutritional quality. PDCAAS is based on fecal digestibility and the proportion of essential amino acids provided by a protein, with a maximum score of 1.0. DIAAS, recommended by the FAO [24], represents a methodological improvement by considering true ileal digestibility and allowing scores above 100, enabling a more precise and differentiated assessment of protein quality.

In this context, quinoa has shown PDCAAS values between 0.85 and 0.93 and DIAAS values above 0.80 in cooked samples [29,30,31]. These values are comparable—or even superior in some cases—to those observed in legumes such as soy (PDCAAS ≈ 0.91) or beans (PDCAAS ≈ 0.75). Although still lower than egg (PDCAAS = 1.00; DIAAS > 100) and milk (PDCAAS = 1.00), quinoa is positioned as one of the best plant-based sources of complete protein.

Moreover, technological processes such as germination, fermentation, enzymatic hydrolysis, and extrusion have been shown to significantly enhance the digestibility and bioavailability of proteins in Andean grains. These treatments reduce antinutritional compounds such as phytates, saponins, and tannins, while promoting partial protein breakdown, facilitating absorption [11,32]. For instance, enzymatic hydrolysis of quinoa has been shown to increase the availability of essential amino acids such as lysine and methionine by over 20% [33].

More recently, emerging strategies have been explored, such as the formation of fibrillar or nano-protein structures that can mimic the structural organization of animal proteins and enhance intestinal absorption by simulating digestible protein features [33,34]. These structures not only optimize technological functionality (emulsification, gelation, texture) but also represent a promising approach to increase the protein efficiency of functional foods based on pseudocereals.

### 2.3. Comparison with Animal Proteins and Other Plant Sources

Compared to animal proteins, Andean grains offer important advantages in terms of bioavailability, amino acid balance, and functional potential, although they have lower absolute protein content per serving [4,21].

When compared with other plant sources such as wheat, maize, or even soy, quinoa and cañihua stand out for their lower allergenic potential, better digestibility, and comprehensive nutritional composition [19,29].

When combined with legume proteins (e.g., peas, chickpeas) or functional starches (such as oats or sweet potato), pseudocereals allow for the development of products with biological value equivalent to lean meat or egg [35,36]. This assertion is supported by quantitative comparisons of essential amino acid content between Andean pseudocereals and high-quality animal proteins, as shown in Table 1.

## 3. Functional Properties Relevant for Animal Protein Replacement

### 3.1. Solubility, Emulsifying, Foaming, and Gelling Capacity

Proteins from Andean grains exhibit excellent solubility in aqueous media, especially albumins and globulins, facilitating their incorporation into liquid matrices such as functional beverages, yogurts, and fermented products [11,37]. This property is favored by their flexible globular structure and high content of polar amino acids in their protein fractions [38].

Regarding emulsifying capacity, quinoa protein extracts have demonstrated the ability to form stable emulsions with small droplet size, even without external emulsifiers, due to their affinity for hydrophobic interfaces [39,40]. These characteristics are critical for formulations such as vegan mayonnaise, mousse-type desserts, or plant-based dairy drinks.

The formation of stable foams is another important functional attribute. Quinoa proteins, under controlled pH conditions, can stabilize air–water interfaces through thermal aggregation, showing results comparable to egg albumin [31,40].

In terms of gelling capacity, quinoa-based protein–polysaccharide systems with xanthan gum or carrageenan have been developed to form thermo-reversible gels suitable for products like flan, plant-based cheeses, and emulsified gels [33,38]. Additionally, recent research has explored quinoa proteins’ ability to self-assemble into ordered fibrillar structures resembling amyloids, allowing the formation of stable three-dimensional networks. This type of structuring, which mimics natural protein assembly processes, enhances the gel’s mechanical and functional properties, enabling their application in protein-enriched functional foods [41].

Overall, quinoa, cañihua, and kiwicha proteins exhibit a broad functional spectrum, including high solubility, emulsifying, foaming, and gelling capacities, enabling their application in liquid, semi-solid, and solid food matrices. These properties are determined by structural factors such as the content and distribution of polar and hydrophobic amino acids, the globular conformation of protein fractions, and their ability to form stable three-dimensional networks. Table 2 summarizes these techno-functional properties, their key mechanisms, and representative applications, supported by multiple recent references for each property.

### 3.2. Functional Performance of Pseudocereals in Structured Food Formulations

The use of Andean pseudocereals like quinoa and cañihua in structured food matrices has gained increasing interest in the food industry due to their complete nutritional profile, technological functionality, and versatility in allergen-free and gluten-free formulations. This section explores their performance in specific applications such as meat analogs, dairy alternatives, and gluten-free bakery products.

#### 3.2.1. Meat Analogs Based on Andean Pseudocereals

Proteins from quinoa and cañihua have proven effective in the development of texturized meat analogs, contributing to the cohesion, juiciness, and firmness of the final product [36,42]. Thanks to their emulsifying and gelling capacity, these pseudocereals allow for the formation of fibrous structures that mimic the texture of animal meat.

Moreover, when combined with legume proteins (such as soy, pea, or lupin) or whole cereals, they enhance textural properties and improve water and fat retention during cooking, resulting in juicier and more thermally stable products [35]. This synergy also improves the essential amino acid profile and digestibility of the final product.

#### 3.2.2. Dairy-Free Products Enriched with Quinoa

In addition to their use in meat systems, Andean pseudocereals have been successfully incorporated into non-dairy fermented matrices. For instance, quinoa-based yogurt-like products have been developed with stable rheological properties, good syneresis resistance, and positive sensory acceptability, comparable to traditional dairy products [43].

In vivo studies have shown that quinoa-based plant yogurts have beneficial effects on intestinal and liver health. In animal models, a significant reduction in biomarkers associated with non-alcoholic fatty liver disease after regular consumption of these products was found [44], highlighting their potential as functional foods for populations with specific metabolic needs.

#### 3.2.3. Applications in Gluten-Free Baking

In the bakery sector, the inclusion of quinoa and cañihua in gluten-free formulations has been shown to improve the structure, texture, and nutritional value of baked goods. These flours provide high-quality proteins, minerals, and dietary fiber, along with a more appealing sensory profile than other gluten-free ingredients with neutral flavor or fibrous texture [45].

The use of extruded quinoa flour at substitution levels of 25–45% in gluten-free bread has resulted in significant increases in crumb cohesiveness, elasticity, and resilience, while maintaining sensory acceptability and specific loaf volume [46]. These improvements are attributed to partial starch gelatinization and controlled protein denaturation during extrusion, which promote gas retention and the formation of a continuous structural network in the absence of gluten.

From a technological standpoint, quinoa flour acts as a multifunctional agent: its protein fractions (mainly globulins and albumins) contribute to water retention, stabilize gas bubbles during fermentation and baking, and improve crumb elasticity [47]. Additionally, the functional starches and soluble fibers present in quinoa and cañihua help achieve optimal dough viscosity, which is essential for volume development and structure in gluten-free baking.

The combination of pseudocereals with other plant-based raw materials, such as tarwi or potato starch, has further optimized elasticity and baking tolerance, producing breads with higher specific volume, retained moisture, and a softer texture [48]. These formulations, beyond their improved technological performance, also deliver an enhanced nutritional profile, with increased protein content, fiber, and essential micronutrients.

Beyond their technological contributions, the inclusion of quinoa and cañihua flours in gluten-free baking formulations can also provide functional benefits. Recent studies have shown that these products contain higher levels of dietary fiber, phenolic compounds, and antioxidant activity compared to formulations made exclusively with refined starches, which may help modulate glycemic response and reduce oxidative stress [45,47,48]. For instance, gluten-free sourdough breads enriched with quinoa, amaranth, rice, and spirulina not only improved their protein profile and sensory properties but also exhibited significantly higher antioxidant capacity, suggesting a potential protective effect against metabolic alterations [47]. Complementarily, the incorporation of pseudocereals in gluten-free bread increases nutritional density and the supply of essential amino acids [45], while vegan gluten-free muffins made with cañihua and tarwi blends show high protein and fiber contents as well as sensory stability, reinforcing their potential as functional foods targeted at specialized diets [48]. Taken together, these findings support the dual functionality of Andean pseudocereals in gluten-free baking: optimizing technological quality while simultaneously delivering bioactive compounds with potential health benefits for the consumer.

**Table 2:** Connection between techno-functional properties of Andean pseudocereals and their main food applications.

Functional Property	Key Mechanism	Food Applications	References
Solubility	Protein dispersion in liquid matrices; high proportion of polar amino acids favors hydration	Functional beverages, supplements, shakes	[37,40]
Emulsifying capacity	Stabilization of oil–water interfaces; affinity of protein fractions for hydrophobic surfaces	Dairy substitutes (milk, yogurt, cheese)	[49,50]
Foaming capacity	Air incorporation and stabilization; thermal aggregation under controlled pH conditions	Baked goods	[33,40]
Gelling capacity	Formation of dense protein networks; amyloid-like fibrillar self-assembly	Meat analogues	[28,33]

### 3.3. Synergistic Combinations (Pseudocereal–Legume, Whole Grains, Fibers, and Emerging Matrices)

Synergistic combinations between Andean pseudocereals and other plant-based raw materials represent an effective strategy to optimize both the nutritional profile and the functional performance of food products. When quinoa (*Chenopodium quinoa*), cañihua (*Chenopodium pallidicaule*), or kiwicha (*Amaranthus caudatus*) is combined with legumes such as pea (*Pisum sativum*), chickpea (*Cicer arietinum*), or tarwi (*Lupinus mutabilis*), limiting amino acids—mainly lysine and methionine—are complemented, thereby increasing the biological value of the blend [39,48].

In practical formulations, this synergy has demonstrated significant improvements in technological quality. For example, vegan gluten-free muffins made with cañihua and tarwi flour blends achieve higher specific volume, better moisture retention, and softer texture compared to conventional gluten-free formulations [48]. Likewise, the co-extrusion of pea proteins with amaranth and oat flour produces stable fibrous textures with good water and fat retention, suitable for meat analogue applications [39].

Beyond the protein profile, these combinations enhance key functional properties such as gelling, emulsification, water-holding, foaming, and protein solubility. The addition of soluble fibers such as inulin and functional starches, or hydrocolloids such as xanthan gum, alginates, or fucoidans, contributes to viscosity stabilization, improved protein network cohesion, and increased physicochemical stability of the final matrix [34,49,50].

In cutting-edge applications such as 3D and 4D food printing, matrices composed of pseudocereal proteins and functional polysaccharides have shown printability, stable three-dimensional structures, and rheological properties tailored for the design of personalized foods for athletes, older adults, or individuals with specific dietary requirements [34,37].

Finally, controlled germination of pseudocereals (addressed in Section 4.1) is employed as a complementary pre-treatment to improve the techno-functional properties of these combinations, increasing solubility, viscosity, and emulsifying capacity, thereby expanding their applicability in next-generation structured matrices [51].

## 4. Processing Technologies and their Impact on Protein Quality

### 4.1. Germination, Fermentation, and Enzymatic Hydrolysis

Germination is a traditional yet scientifically validated technique that activates endogenous enzymes such as proteases and phytases, reduces antinutritional factors (phytates, saponins, tannins), and increases the concentration of free amino acids, B-complex vitamins, and phenolic compounds [7,51]. Improvements in protein solubility and functionality after germination have also been observed [52]. Controlled germination of quinoa and cañihua has been shown to increase lysine and arginine content by up to 25%, while improving protein solubility and emulsification capacity [7]. Mechanistically, phytase activity during sprouting hydrolyzes phytic acid, improving mineral bioavailability and enhancing protein digestibility [53].

Fermentation—either spontaneous or with defined cultures such as *Lactobacillus plantarum* or *Bacillus subtilis*—promotes proteolysis through microbial and endogenous enzymes, releasing low-molecular-weight bioactive peptides with antioxidant, antihypertensive, and anti-inflammatory activities [54,55]. These transformations improve protein digestibility and also improve water-holding and emulsifying capacities, making fermented pseudocereal matrices suitable for dairy-free yoghurts, plant-based cheeses, and functional beverages.

Moreover, fermentation can induce the activation of endogenous enzymes in the grains themselves—such as phytases, amylases, and proteases—which complement the biochemical transformations promoted by microorganisms [53].

Enzymatic hydrolysis, using proteases such as alcalase or flavourzyme, produces low-molecular-weight peptides that enhance foaming and gelling capacities, while increasing the availability of essential amino acids like lysine and methionine [32]. Such modifications are particularly relevant for high-protein beverages, mousse-like desserts, and protein gels targeting populations with specific nutritional needs.

### 4.2. Thermal and Non-Thermal Processing Technologies

Modern technologies applied to Andean grains enable the modification and enhancement of their physical, functional, and nutritional properties. Among the most commonly used are thermal extrusion, micronization, and emerging technologies such as high-pressure processing.

#### 4.2.1. Extrusion

Thermal extrusion is widely used to transform the physicochemical properties of Andean grains. This process combines high temperature, pressure, and mechanical shear, causing starch gelatinization, protein denaturation, Maillard reactions, and a reduction in antinutritional factors like saponins and phytates. It also promotes the formation of disulfide bonds and complexes among starch, proteins, and lipids that improve the final product’s technological functionality [56,57]. These transformations yield ingredients with greater digestibility, solubility, water-holding capacity, emulsification, and structural cohesion.

In cañihua–rice blends, extrusion has generated expanded products with good sensory acceptability and improved nutritional values [58]. In extruded snacks and cereals based on quinoa and amaranths, improved protein digestibility and a significant reduction in antinutrients have been demonstrated [59].

Moreover, recent studies have shown that using extruded quinoa flour in gluten-free bread formulations improves both nutritional quality and technological functionality. Particularly, when extruded quinoa flour obtained using two extrusion conditions (up to 139 °C and 145 °C) was used at 25–45% substitution levels, there were observed increases in bread protein, dietary fiber, and ash content, along with improvements in crumb cohesiveness, elasticity, and resilience [46]. The flour submitted to the more drastic treatment maintained sensory acceptability and specific loaf volume, demonstrating that extrusion can produce functional ingredients suitable for gluten-free products.

Wet extrusion, on the other hand, allows for the development of meat analogs with fibrous structure and texture similar to real meat products and functional protein profiles [60]. Pre-treatments such as fermentation or partial hydrolysis enhance the textural and sensory results of these products [61], broadening the applications of Andean pseudocereals in vegetarian formulations.

#### 4.2.2. Micronization and Cryogenic Milling

To improve dispersibility, solubility, and functional performance of pseudocereal flours in food matrices, particle size reduction techniques such as micronization and cryogenic milling have been applied. These methods alter grain surface morphology and allow for more homogeneous distribution in bakery or beverage products. Micronization is a mechanical ultra-fine grinding process that reduces particles to sizes below 100 µm, increasing specific surface area and enhancing interactions with water and other components. In quinoa flours, this has translated into better emulsifying capacity, improved rheology, and increased mineral bioavailability [21].

Cryogenic milling uses refrigerant gases like liquid nitrogen to cool the material before and during grinding. This method prevents thermal degradation of sensitive compounds such as phenolics and peptides while achieving finer particle distribution for consistent quality and enabling more controlled fragmentation. These techniques have shown to improve emulsifying capacity, rheology, and mineral bioavailability in quinoa flours [62].

#### 4.2.3. Atmospheric Cold Plasma (ACP)

Atmospheric cold plasma (ACP) has also been explored as a non-thermal alternative to improve techno-functional properties of Andean flours and reduce microbial load without affecting nutritional value. This technology generates a mixture of reactive species (radicals, ions, electrons, ozone) from gases such as air or argon, exposed to an electric field at room temperature and atmospheric pressure. ACP acts on the surface of foods, inducing structural changes and enhancing functionality without thermal damage. In quinoa flours, cold plasma treatment has been reported to improve water absorption, viscosity, and foaming capacity while preserving the bioactivity of functional peptides. Moreover, it contributes to effective microbial reduction, extending product shelf life without the need for preservatives [62]. Similar benefits have been reported in amaranth starch, with enhanced functional performance for bakery and beverage formulations [63].

In quinoa flour, dielectric barrier discharge (DBD) atmospheric cold plasma has been associated with increases in water absorption capacity and changes in the pasting profile, which is consistent with a mild reorganization of starch and proteins [62,64]. On the other hand, ACP applied to quinoa protein isolates led to an increased protein solubility and improved foaming and emulsifying activity, promoting stabilizing interactions (e.g., disulfide bridges) without deteriorating nutritional value [65]. In another study, the use of ACP in amaranth (kiwicha) starch caused changes in crystallinity, reduced syneresis, and increased solubility and water uptake. These changes have led to better stabilizer performance in low-fat systems [63]. Overall, recent quinoa reviews emphasize that ACP can enhance functionality and contribute to safety without heat damage, provided that treatment time/voltage and gas atmosphere are properly controlled [64,66].

#### 4.2.4. High-Pressure Processing 

High-pressure processing (HPP) is another non-thermal technology recently applied to flours, suspensions, and pastes made from Andean grains. This method improves microbiological quality and technological functionality without compromising nutritional profiles. It involves subjecting food to high hydrostatic pressures (typically 300–600 MPa) for short durations (1–10 min) under isothermal or low-temperature conditions.

In pseudocereals such as quinoa or kiwicha, HPP can induce reversible structural changes in proteins, promoting partial unfolding and increasing exposure of functional groups (e.g., sulfhydryl and carboxyl), thereby improving gelling, emulsifying, and water-holding capacities. HPP also enhances protein digestibility and reduces residual antinutritional enzymatic activity, making it highly suitable for vegetarian, gluten-free, or high-functionality food products [67,68]. Additionally, it offers benefits in food safety and shelf life by significantly reducing microbial load in flours and mixtures without the need for severe thermal treatments or additives. This makes it particularly valuable for ready-to-eat, and clean-label products.

In quinoa flour, HPP up to 600 MPa modifies physicochemical and gelatiniza-tion/pasting properties, which have repercussions on gel texture [69]. Similarly, combining HPP with different pH reorganizes the secondary structure of quinoa protein isolates, increases their solubility, and allows for the achievement of protein isolates with tailored properties, thereby optimizing their functional performance [70]. For amaranth proteins, high-pressure pretreatment promotes cross-linking and film formation with enhanced mechanical and barrier properties, highlighting the versatility of HPP for plant-protein matrices [67].

Furthermore, when germinated quinoa flour was submitted to HPP (200–500 MPa), improved functional, rheological, and microstructural attributes were observed, yielding gluten-free matrices with better structure [71]. On the other hand, the application of a HPP treatment ranging from 300 to 600 MPa to quinoa flour/water dispersions (1:2) led to structural, functional, and antioxidant changes depending on the intensity of the HPP. For instance, a complete starch gelatinization was observed at 600 MPa [72].

Thermal and non-thermal technologies applied to Andean pseudocereals modify their protein and starch structures, enhancing functional properties such as solubility, gelling capacity, water-holding ability, and microbiological stability. These effects depend on processing conditions and the intrinsic characteristics of each raw material. Table 3 provides a summary of the main evaluated technologies, the observed functional and nutritional benefits, application examples, and the corresponding references supporting each case.

## 5. Applications in Vegan and Vegetarian Product Formulations

### 5.1. Meat Analogs and Dairy Substitutes

Andean pseudocereals are being actively integrated into the development of meat analogs, taking advantage of their ability to form fibrous structures, strong protein cohesion, and high nutritional value. Formulations based on quinoa, cañihua, or kiwicha combined with soy or pea have shown significant improvements in texture, water retention, and sensory profiles [36,73]. Recent studies demonstrate that Andean proteins improve the rheological matrix of meat analogs when used as structural complements [74,75].

In the development of plant-based dairy analogs, quinoa has been used in the formulation of fermented beverages, yogurts, and cream-type cheeses, with excellent results in terms of viscosity, sensory acceptability, and colloidal stability [43,76]. Particularly, the development of fermented cheeses using water kefir and pseudocereals has significantly improved the organoleptic profile, resulting in soft textures and pleasant aromas [76,77]. These fermented matrices represent a promising alternative in the “plant-based” category. Additionally, it has been shown that high-protein beverages made from amaranth exhibit stable functional structures during simulated gastrointestinal digestion and can release peptides with potential antihypertensive activity, reinforcing their value as functional plant-based foods [78].

### 5.2. Cereal-Based Products: Pasta, Bread, Breakfast Cereals

For the development of bakery products, Andean grains offer excellent functionality for gluten-free products, as they provide structure, partial elasticity, and improved nutritional profiles. Breads formulated with blends of quinoa, rice, and spirulina have shown good expansion, balanced protein content, and high acceptability [47]. Additionally, the use of cañihua has resulted in breads with higher specific volume, better moisture retention, and softer textural characteristics [79].

Likewise, the use of cañihua and tarwi flours in fermentable matrices has enabled the production of gluten-free vegan products with good specific volume, appropriate moisture, and acceptable textural properties [48]. Since these flours have high water and oil absorption capacities, when incorporated into muffin batters, they contribute to forming a complex structure rich in protein and fiber capable of retaining water and stabilizing air bubbles during baking. These properties improve both texture and the nutritional profile of the final product, highlighting the potential of Andean pseudocereals in plant-based fermented and baked goods.

More recently, it was found that the partial substitution of wheat flour with quinoa and tarwi flours in breadmaking enhanced dough extensibility, improved crumb cohesion, and maintained acceptable volume and texture [37]. These findings confirm the technological viability of pseudocereal–legume blends in cereal-based bakery products aimed at functional and plant-based markets.

In pasta and breakfast cereals, hyperprotein quinoa flour has been used in formulations with proper textures, optimal cooking times, and good storage stability [59,80]. Cookies, energy bars, and extruded cereals formulated with pseudocereal blends have also been positively evaluated in sensory and nutritional studies, confirming their value in the development of healthy and appealing products for modern consumers [81,82].

### 5.3. Consumer Acceptance, Textural and Sensory Properties

The interest in developing vegan and vegetarian foods has driven the incorporation of Andean pseudocereals into a wide variety of food matrices (Table 4). The functional and nutritional properties of quinoa, cañihua, amaranth, and kiwicha have enabled the formulation of products that not only meet industrial processing requirements but also show high levels of consumer acceptance.

Although there are emerging commercial initiatives using Andean pseudocereals in vegan and vegetarian formulations, the availability of consolidated brands and products in the global market remains limited [64,66]. Furthermore, no systematic studies have been found that characterize the market penetration or dynamics of these products. For this reason, and to maintain an academic and evidence-based approach, this review focuses on describing the technological and nutritional applications documented in the scientific literature, avoiding direct references to specific brands.

In plant-based meat products such as burgers, combining quinoa with pea protein has resulted in fibrous structures with good texture and balanced sensory profiles, facilitating their use as plant-based analogs [36]. In the segment of fermented plant-based dairy products, quinoa-based yogurts have demonstrated stable viscosity and good sensory acceptability [43], while cream-type cheeses formulated with amaranth fermented with water kefir offer smooth textures and organoleptic profiles similar to commercial dairy products [76]. These applications are summarized in Table 5, highlighting how Andean pseudocereals can be successfully integrated into various functional categories.

In addition to the applications documented in scientific studies, experiences from regional product development initiatives suggest that integrating Andean pseudocereals into mainstream food matrices also demands a careful balance between nutritional goals and sensory expectations. For instance, the use of whole-grain flours from cañihua or mashua may result in darker color tones or earthy flavor notes that require thoughtful formulation strategies, especially in markets not accustomed to these native grains.

These considerations highlight the need to involve consumer education, storytelling around ancestral foods, and iterative sensory testing to facilitate acceptance and trust. While scientific literature supports the nutritional and functional promise of these ingredients, their real-world implementation benefits greatly from sociocultural contextualization and inclusive product design.

In bakery products, using blends such as quinoa and spirulina has produced gluten-free breads with good volume, higher protein content, and adequate texture [47]. Similarly, the incorporation of hyperprotein quinoa in pasta has improved elasticity, cooking time, and the structural quality of the final product [80]. Extruded snacks developed with quinoa and cañihua blends offer crunchy texture, shelf-life stability, and suitable nutritional profiles for modern consumers [59]. Additionally, functional beverages based on kiwicha and oats have been formulated with high fiber content and free from common allergens, aligning with clean label trends and restrictive diets [81].

## 6. Sustainability and Socioeconomic Impacts

### 6.1. Environmental Footprint (Greenhouse Gas Emissions, Water Use, and Land Use)

The environmental footprint of protein sources varies considerably depending on the production system, geographic origin, and processing efficiency. Among animal-derived proteins, beef shows the highest impact, with 27.0 kg CO_2_-eq and 15,400 L of water per kilogram of protein, followed by pork (12.1 kg CO_2_-eq; 6000 L), chicken (6.9 kg CO_2_-eq; 4300 L), fish (5.4 kg CO_2_-eq; 3900 L), and cow’s milk (3.2 kg CO_2_-eq; 1020 L). Plant-based proteins such as soy (2.0 kg CO_2_-eq; 2145 L), pea (2.2 kg CO_2_-eq; 2050 L), and wheat (2.7 kg CO_2_-eq; 1800 L) display substantially lower values. In this context, Andean pseudocereals—quinoa (0.9 kg CO_2_-eq; 1200 L) and cañihua (0.7 kg CO_2_-eq; 850 L)—stand out for having the lowest greenhouse gas emissions and water requirements in this comparison. Table 6 summarizes these data obtained from recent peer-reviewed life cycle assessments [85,86,87].

### 6.2. Contribution to Agrobiodiversity and Food Security

Quinoa, cañihua, and kiwicha belong to a group of resilient native crops from the Andes, recognized for their genetic diversity, ecological adaptability, and high nutritional value. Their cultivation promotes the conservation of local agrobiodiversity and the preservation of traditional agroecological systems that have endured for millennia [88,89].

Quinoa, for example, comprises over 3000 local varieties adapted to various altitudinal zones ranging from 2500 to over 4000 m above sea level, with tolerance to saline soils, drought, and frost [87]. This versatility allows its inclusion in climate adaptation strategies, especially in marginal areas or regions vulnerable to desertification [90].

From a food security perspective, these crops are crucial for ensuring access to high-quality protein in regions where animal sources are limited or expensive. Their integration into food and nutrition security programs has shown positive outcomes in reducing deficiencies in iron, zinc, and essential amino acids, particularly in children and pregnant women [91,92].

### 6.3. Potential for Local Economies and the Circular Bioeconomy

In addition to their environmental role, Andean pseudocereals have a direct impact on rural economies, especially in countries such as Peru, Bolivia, and Ecuador. The commercial valorization of these crops has contributed to the economic empowerment of farming communities through rural employment generation, the strengthening of fair trade, and the export of value-added products [88,89].

Several studies have emphasized that promoting value chains based on Andean grains stimulates the circular bioeconomy by reusing agro-industrial by-products such as quinoa husks, milling residues, or bagasse for animal feed, edible biofilm production, and cosmetic formulations [93]. This approach reduces losses, adds value, and promotes comprehensive sustainability across the agri-food system.

This production model not only optimizes the use of local resources but also offers clear environmental advantages over other protein sources. For instance, Table 6 shows that quinoa and cañihua generate between 0.7 and 0.9 kg of CO_2_-equivalent per kilogram of protein—far below the 27 kg for beef or 3.2 kg for cow’s milk. Likewise, their water consumption does not exceed 1200 L per kilogram, in stark contrast to the over 15,000 L required for beef production. These data position Andean grains as strategic ingredients for resilient, low-carbon, and resource-efficient food systems.

Moreover, the recovery of ancestral knowledge related to the cultivation, processing, and ceremonial use of these grains contributes to sociocultural sustainability, a key element for achieving the Sustainable Development Goals (SDGs), particularly Goals 2 (Zero Hunger), 12 (Responsible Consumption and Production), and 15 (Life on Land).

## 7. Challenges, Limitations, and Future Perspectives

### 7.1. Technological Barriers: Scaling up, Functionalization, and Formulation

Based on reviewed studies and the observed limitations reported in pilot applications across Andean countries, it becomes evident that the industrial scalability of pseudocereals such as quinoa, cañihua, and kiwicha is hindered by multiple technical and contextual factors. For example, differences in post-harvest handling, such as variability in desaponification efficacy or particle size standardization, directly affect product consistency and functionality.

Several authors coincide in highlighting that specific textural and sensory challenges arise in gluten-free formulations when pseudocereals are used as the primary structure-forming ingredient. From a practical standpoint, local product development efforts have shown that even small differences in cultivar, altitude of cultivation, or milling conditions can lead to significant variations in dough rheology and consumer acceptance.

Therefore, the current literature supports the notion that future scaling strategies should not only rely on advanced processing technologies, but also integrate local know-how, targeted sensory optimization, and regulatory adaptation for functional labeling.

Although the nutritional and functional value of Andean pseudocereals is well-documented, there are significant challenges in their industrial scaling and technological standardization. The main barriers include genetic variability among local varieties, differences in post-harvest processing, and the lack of technologies adapted to the specific rheological and sensory properties of these grains [36,59].

Formulating complex products such as meat or dairy analogs requires precise control of protein–water–lipid interactions, which is not always easily achievable with unmodified pseudocereals. As result, techniques such as wet extrusion, blending with functional legume proteins, and incorporating polysaccharides are used to improve the texture, cohesion, and juiciness of these products [40,74].

Likewise, standardization of processing steps such as desaponification, germination, and fermentation is essential to ensure consistent sensory and biofunctional quality, especially in large-scale production contexts.

### 7.2. Nutritional, Regulatory, and Consumer Perception Aspects

Although quinoa, cañihua, and kiwicha possess excellent nutritional profiles, more clinical studies are needed to validate their metabolic effects in humans—especially in comparison with animal proteins. Additionally, the presence of residual antinutrients (phytates, oxalates, saponins) can limit mineral and amino acid absorption if appropriate treatments are not applied [54,86].

From a regulatory standpoint, the legal recognition of pseudocereals as functional ingredients or novel foods varies by region. While in Europe certain extracts or fractions are regulated by European Food Safety Authority (EFSA), in Latin America there is still fragmented classification and risk assessment [94]. This hinders their widespread use in industrialized products with nutritional or functional claims.

Consumer acceptance may also be affected by the bitter taste of some quinoa varieties, or by limited awareness of cañihua and kiwicha outside their traditional consumption areas [95,96]. The classification of products as “meat analogs” or “dairy substitutes” also shapes consumer expectations, posing a challenge for strategic design of labels, messaging, and product formats [95].

### 7.3. Emerging Strategies: Nanotechnology, Bioactive Peptides, and Clustered Regularly Interspaced Short Palindromic Repeats (CRISPR) Gene Editing

Emerging technologies represent a promising pathway to maximize the use of Andean pseudocereals. The application of food nanotechnology—such as encapsulating quinoa proteins or bioactive compounds in starch or lipid nanoparticles—has shown improvements in stability, intestinal absorption, and sensory functionality [34,41].

Likewise, the generation of bioactive peptides through controlled enzymatic hydrolysis has shown potential for functional food applications with antihypertensive, antioxidant, and antidiabetic effects, especially in cañihua and amaranth extracts [54,55]. Recent evidence indicates that a high-protein beverage based on amaranth generates antihypertensive peptides after simulated gastrointestinal digestion, underscoring this pseudocereal’s value in next-generation foods [78].

Finally, gene editing techniques such as CRISPR-Cas9 are emerging as tools to improve protein content, reduce antinutrients, or standardize technological quality in pseudocereals. However, there are still considerable ethical, regulatory, and technical limitations to their application in foods intended for mass consumption [94].

## 8. Conclusions

Andean grains—particularly quinoa, cañihua, and kiwicha—represent an exceptional source of plant-based proteins with high nutritional value and remarkable technological functionality, and their use contributes significantly to environmental sustainability. Throughout this review, it has been demonstrated that these pseudocereals offer a complete profile of essential amino acids, digestibility comparable to animal proteins, and outstanding functional versatility that enables their incorporation into products such as meat analogs, dairy substitutes, baked goods, and functional beverages.

From an environmental perspective, their cultivation results in low greenhouse gas emissions, reduced water requirements, and minimal agricultural land use, positioning them as key ingredients in the transition toward sustainable, climate-resilient food systems. Their production supports the preservation of agrobiodiversity, local food security, and the strengthening of rural Andean economies, simultaneously promoting ecological, economic, and sociocultural sustainability.

Nevertheless, technological and regulatory limitations remain to be addressed. Among them are industrial scaling, the need for standardized processing and desaponification methods, and regulatory gaps in recognizing these ingredients across different markets. Additionally, consumer perception—especially outside the Andean region—requires more effective communication, labeling, and product positioning strategies.

Emerging research avenues open new opportunities to further enhance their potential: the application of advanced technologies such as food nanotechnology, the generation of functional bioactive peptides, and precision gene editing could lead to substantial improvements in functionality, stability, and nutritional profiles. However, these approaches require scientific validation, risk assessment, and the development of clear regulatory frameworks.

Unlike previous reviews on Andean pseudocereals, this work systematically integrates nutritional, functional, and technological aspects, with particular emphasis on emerging technologies (such as 3D and 4D food printing, protein and bioactive compound encapsulation, and CRISPR-Cas9 gene editing) and their potential application in non-allergen- and gluten-free matrices. It also includes expanded quantitative comparisons of amino acid profiles and environmental footprints against conventional animal and plant proteins, providing a comprehensive and up-to-date perspective on their role as strategic substitutes for animal protein in vegetarian and vegan diets.

In summary, Andean pseudocereals possess the essential attributes to be established as strategic substitutes for animal protein in vegetarian and vegan diets, contributing to the development of healthier, more sustainable, and inclusive foods. Their valorization through science, technological innovation, and sociocultural integration will be key to their global positioning and to achieving multiple Sustainable Development Goals (SDGs).

## Figures and Tables

**Table 1:** Approximate content of essential amino acids (mg/100 g dry product) and crude protein (%) in Andean pseudocereals and in selected animal and plant proteins.

Protein Source	Crude Protein (%)	Lysine	Methionine	Threonine	Leucine	Isoleucine	Valine	Phenylalanine	Tryptophan	Histidine	Reference
Quinoa	16.3	580	290	320	840	430	580	660	90	250	[9,21]
Cañihua	15.8	610	310	340	870	450	600	690	95	260	[19]
Kiwicha	15.0	540	270	300	810	410	570	640	88	240	[20]
Egg	12.6	930	370	490	1080	670	760	690	170	270	[22]
Beef	21.0	880	260	470	1740	800	990	890	220	390	[23]
Milk	3.3	760	260	430	1010	590	650	510	140	240	[21,24]
Wheat	12.0	260	150	280	660	300	400	480	110	180	[21]
Rice	7.0	300	120	280	610	250	390	450	100	150	[21]
Maize	9.0	270	150	270	730	270	390	420	60	150	[21]
Tarwi (*Lupinus mutabilis*)	42.0	620	210	370	800	370	450	510	120	210	[25]
Pajuro (*Erythrina edulis*)	21.5	590	180	350	770	360	430	490	110	200	[26]
Sacha inchi (*Plukenetia volubilis*)	27.0	640	230	380	810	370	460	530	115	220	[27]

Note: In the case of pajuro (*Erythrina edulis*), the amino acid values were calculated from data reported in g/100 g protein by [26], using the crude protein content on a dry basis reported in the same study to convert them to mg/100 g dry product.

**Table 3:** Impact of processing technologies on Andean grains.

Technology	Functional Benefit	Nutritional Improvement	Application Examples	References
Extrusion (dry/wet)	Increases texture and expansion, reduces bulk density; formation of fibrous structures in wet extrusion	Enhances protein digestibility, reduces antinutrients (phytates, saponins), improves crumb cohesion and elasticity	Gluten-free breads, expanded snacks, meat analogues	[46,56,57,61]
Micronization/Cryogenic milling	Improves rheology, hydration capacity, homogeneous particle distribution, and colloidal stability	Increases mineral bioavailability, preserves heat-sensitive compounds	Ready-to-drink beverages, gluten-free bakery	[21]
Atmospheric cold plasma (ACP)	Improves water absorption, viscosity, and foaming capacity; reduces microbial load	Minimal loss of bioactive compounds, preservation of functionality	Clean-label formulations, bakery, functional beverages	[62,63]
High-pressure processing (HPP)	Improves gelling, water-holding, and emulsifying capacities, enhances microbiological stability	Increases protein digestibility, reduces antinutritional enzymatic activity, minimal nutritional loss	Ready-to-eat products, gluten-free, high-functional-value matrices	[67,70]

**Table 4:** Sensory evaluation and acceptability of pseudocereal-based products.

Evaluated Product	Evaluation Method	Main Findings	Reference
Bread with cañihua flour	Sensory panel	Higher specific volume, neutral flavor	[79]
Yogurt with fermented quinoa	9-point acceptance test	High acceptability in texture and flavor	[43]
Extruded quinoa snacks	Trained panel	Good crunchiness, scores ≥ 7/9	[83]
Vegan cheese with kefir	Texture profile	Texture comparable to commercial cream cheese	[76]
Perception survey	Consumers (n = 250)	High purchase intent for pseudocereal-based products	[84]

Note: All evaluations refer to gluten-free, allergen-free, and clean-label formulations.

**Table 5:** Applications of Andean pseudocereals in vegan and vegetarian formulations.

Product	Grain Used	Key Application	Reference
Plant-based burger	Quinoa + pea	Fibrous structure, balanced sensory profile	[36]
Fermented plant yogurt	Quinoa	Stable viscosity and good acceptability	[43]
Vegan cream cheese	Amaranth + kefir	Smooth texture, favorable fermentation profile	[76]
Gluten-free bread	Quinoa + spirulina	Good expansion, higher protein content	[47]
Functional pasta	Hyperprotein quinoa	Improved cooking properties and elasticity	[80]
Extruded snack	Quinoa + cañihua	Crunchy texture, good storage stability	[59]
Functional plant drink	Kiwicha + oats	High fiber content, free from common allergens	[81]

**Table 6:** Environmental footprint comparison among protein sources (per kg of protein).

Protein Source	GHG Emissions (kg CO_2_-eq/kg)	Water Use (L/kg)	References
Beef	27.0	15,400	[85]
Pork	12.1	6000	[85]
Chicken	6.9	4300	[85]
Fish	5.4	3900	[85]
Cow’s milk	3.2	1020	[85]
Soy protein	2.0	2145	[86]
Pea protein	2.2	2050	[86]
Wheat	2.7	1800	[86]
Quinoa	0.9	1200	[87]
Cañihua	0.7	850	[87]

GHG: greenhouse gas emissions.

## Data Availability

No new data were created or analyzed in this study. Data sharing is not applicable to this article.

## Abbreviations

The following abbreviations are used in this manuscript:

PDCAAS	Protein Digestibility-Corrected Amino Acid Score
DIAAS	Digestible Indispensable Amino Acid Score
BCAA	Branched-Chain Amino Acids
HPP	High-Pressure Processing
ACP	Atmospheric Cold Plasma
CRISPR	Clustered Regularly Interspaced Short Palindromic Repeats
FAO	Food and Agriculture Organization of the United Nations
SDG	Sustainable Development Goal
EFSA	European Food Safety Authority

## References

[1] Wang M., Zhao R. (2023). A Review on Nutritional Advantages of Edible Mushrooms and Its Industrialization Development Situation in Protein Meat Analogues. J. Future Foods.

[2] Xavier J.R., Shashikumar S.H., Vats D., Chauhan O.P. (2025). Future Trends in Plant-Based Meat: Consumer Perception, Market Growth and Health Benefits. Future Foods.

[3] Yeo M.T.Y., Bi X., Henry C.J. (2023). Are Plant-Based Meat Analogues Richer in Minerals than Their Meat Counterparts?. Food Humanit..

[4] Das D., Sheikh M.A., Mir N.A. (2025). Exploring the Potential of Amaranth Proteins: Composition, Functional Characteristics, Modifications, Bioactive Peptides, and Possible Applications in Food and Packaging Industries. J. Food Compos. Anal..

[5] Ihsan A., Ahmad Z., Zheng J., Bilal M., Muhammad Rizwan Abid H., Hu A. (2024). New Trends in Functionalities and Extraction of Plant Proteins in Designing Plant-Based Meat Analogues: A Critical Review. Food Biosci..

[6] Sakai K., Ravishankar G.A., Ranga Rao A., Tahergorabi R., Mohan A.B.T.-H. (2024). Chapter 17—Functional Properties of Meat Analog Products Consisting of Plant-Derived Proteins. Handbook of Plant-Based Meat Analogs.

[7] Lan Y., Wang X., Wang L., Zhang W., Song Y., Zhao S., Yang X., Liu X. (2024). Change of Physiochemical Characteristics, Nutritional Quality, and Volatile Compounds of *Chenopodium quinoa* Willd. during Germination. Food Chem..

[8] Mérida-López J., Rojas C.C., Bergenståhl B., Purhagen J. (2024). Functional Properties of Starch Cultivars of Two Andean Grains Grown in Bolivia: Amaranth (*Amaranthus caudatus*) and Canihua (*Chenopodium pallidicaule*). Heliyon.

[9] Mota C., Santos M., Mauro R., Samman N., Matos A.S., Torres D., Castanheira I. (2016). Protein Content and Amino Acids Profile of Pseudocereals. Food Chem..

[10] Chmelík Z., Šnejdrlová M., Vrablík M. (2019). Amaranth as a Potential Dietary Adjunct of Lifestyle Modification to Improve Cardiovascular Risk Profile. Nutr. Res..

[11] Hadidi M., Aghababaei F., Mahfouzi M., Zhang W., Julian McClements D. (2024). Amaranth Proteins: From Extraction to Application as Nanoparticle-Based Delivery Systems for Bioactive Compounds. Food Chem..

[12] Li G., Chen J., Zhu F. (2023). Comparative Study of Rheological Properties and Pickering Emulsion Stabilizing Capacity of Nonenyl Succinic Anhydride and Octenyl Succinic Anhydride Modified Amaranth Starches. Int. J. Biol. Macromol..

[13] Sanz-Penella J.M., Wronkowska M., Soral-Smietana M., Haros M. (2013). Effect of Whole Amaranth Flour on Bread Properties and Nutritive Value. LWT.

[14] Zahari I., Rinaldi S., Ahlstrom C., Östbring K., Rayner M., Purhagen J. (2023). High Moisture Meat Analogues from Hemp—The Effect of Co-Extrusion with Wheat Gluten and Chickpea Proteins on the Textural Properties and Sensorial Attributes. LWT.

[15] Huang R., Huang K., Guan X., Li S., Cao H., Zhang Y., Lao X., Bao Y., Wang J. (2021). Effect of Defatting and Extruding Treatment on the Physicochemical and Storage Properties of Quinoa (*Chenopodium quinoa* Wild) Flour. LWT.

[16] Peng S., Li Y., Liu H., Tuo Y., Dang J., Wang W., You H., Du S., Wang L., Ding L. (2024). Influence of Germination and Roasting on the Characteristic Volatile Organic Compounds of Quinoa Using Sensory Evaluation, E-Nose, HS-GC-IMS, and HS-SPME-GC-MS. Food Chem. X.

[17] Tiga B.H., Kumcuoglu S., Vatansever M., Tavman S. (2021). Thermal and Pasting Properties of Quinoa—Wheat Flour Blends and Their Effects on Production of Extruded Instant Noodles. J. Cereal Sci..

[18] Olagunju A.I., Omoba O.S., Lorenzo J.M., Bangar S.P. (2023). Amaranth Starch: Physicochemical, Functional, and Nutritional Properties. Non-Conventional Starch Sources.

[19] Moscoso-Mujica G., Mujica Á., Chura E., Begazo N., Jayo-Silva K., Oliva M. (2024). Kañihua (*Chenopodium pallidicaule* Aellen), an Ancestral Inca Seed and Optimal Functional Food and Nutraceutical for the Industry: Review. Heliyon.

[20] Martinez-Lopez A., Millan-Linares M.C., Rodriguez-Martin N.M., Millan F., Montserrat-de la Paz S. (2020). Nutraceutical Value of Kiwicha (*Amaranthus caudatus* L.). J. Funct. Foods.

[21] Nandan A., Koirala P., Dutt Tripathi A., Vikranta U., Shah K., Gupta A.J., Agarwal A., Nirmal N. (2024). Nutritional and Functional Perspectives of Pseudocereals. Food Chem..

[22] Puglisi M.J., Fernandez M.L. (2022). The Health Benefits of Egg Protein. Nutrients.

[23] Orkusz A. (2021). Edible Insects versus Meat—Nutritional Comparison: Knowledge of Their Composition Is the Key to Good Health. Nutrients.

[24] FAO (2013). Dietary Protein Quality Evaluation in Human Nutrition: Report of an FAO Expert Consultation, 31 March–2 April 2011, Auckland, New Zealand.

[25] Salazar D., Arancibia M., Silva D.R., López-Caballero M.E., Montero M.P. (2021). Exploring the Potential of Andean Crops for the Production of Gluten-Free Muffins. Agronomy.

[26] Intiquilla A., Jiménez-Aliaga K., Zavaleta A.I., Arnao I., Peña C., Chávez-Hidalgo E.L., Hernández-Ledesma B. (2016). Erythrina Edulis (Pajuro) Seed Protein: A New Source of Antioxidant Peptides. Nat. Prod. Commun..

[27] Bocanegra Morales N., Galeano Garcia P. (2023). Chemical Composition, Fatty Acid Profile, and Optimization of the Sacha Inchi (*Plukenetia volubilis* L.) Seed-Roasting Process Using Response Surface Methodology: Assessment of Oxidative Stability and Antioxidant Activity. Foods.

[28] Zhang L., Langlois E., Williams K., Tejera N., Omieljaniuk M., Finglas P., Traka M.H. (2024). A Comparative Analysis of Nutritional Quality, Amino Acid Profile, and Nutritional Supplementations in Plant-Based Products and Their Animal-Based Counterparts in the UK. Food Chem..

[29] Jan N., Hussain S.Z., Naseer B., Bhat T.A. (2023). Amaranth and Quinoa as Potential Nutraceuticals: A Review of Anti-Nutritional Factors, Health Benefits and Their Applications in Food, Medicinal and Cosmetic Sectors. Food Chem. X.

[30] Kumar R., Bhardwaj S., Sikarwar M., Kumar A., Singh B.R., Gupta M., Shukla R. (2025). Innovative Food Applications of Quinoa: Exploring Novel Insights into Its Nutritional Potential and Biological Activities. J. Food Compos. Anal..

[31] Zhu F. (2023). Amaranth Proteins and Peptides: Biological Properties and Food Uses. Food Res. Int..

[32] Polo Muñoz M.P., Pismag Portilla R.Y., Bravo Gomez J.E., Solanilla Duque J.F., Roa Acosta D.F. (2025). Instant Quinoa Powder: Effect of Enzymatic Hydrolysis and Extrusion on Its Physicochemical and Rheological Properties. NFS J..

[33] Chen L., Zhang C., Yu X., Fu L., Tang X., Feng X. (2025). Enhance Quinoa Protein Foaming Properties Through Amyloid-like Fibrillation and 2S Albumin Blending. Food Hydrocoll..

[34] Pereira N.I.A., dos Santos Oliveira M., Reis B.C.C., Nascimento B.L., Carneiro C.R., Arruda T.R., Vieira E.N.R., de Castro Leite Junior B.R. (2024). Unconventional Sourced Proteins in 3D and 4D Food Printing: Is It the Future of Food Processing?. Food Res. Int..

[35] Gaber S.M., Dessev T., Knezevic D., Haustveit G., Knutsen S.H. (2025). A Feasibility Study on Oat Concentrates as a Single Source of Protein for Production of Meat Analogues. LWT.

[36] Sharma M., Kaur I., Kumar P., Verma A.K., Umaraw P., Mehta N., Ismail-Fitry M.R., Sharma N., Sazili A.Q. (2024). Processing of Plant Proteins in the Development of Plant-Based Meat Analogs. Handbook of Plant-Based Meat Analogs: Innovation, Technology and Quality.

[37] Vidaurre-Ruiz J., Bender D., Schönlechner R. (2023). Exploiting Pseudocereals as Novel High Protein Grains. J. Cereal Sci..

[38] Zhao K., Hao Y., Guo X., Chang Y., Shen X. (2024). Development, Characterization and Underling Mechanism of 3D Printable Quinoa Protein Emulsion Gels by Incorporating of Different Polysaccharides for Curcumin Delivery. Int. J. Biol. Macromol..

[39] González-Galeana C., Castañeda-Salazar A., del Cortez-Trejo M., Gaytán-Martínez M., Campos-Vega R., Mendoza S. (2025). Structural and Functional Properties of a High Moisture Extruded Mixture of Pea Proteins (*Pisum sativum*), Amaranth Flour (*Amaranthus hypochondriacus*), and Oat Flour (*Avena sativa*). Food Chem..

[40] Van de Vondel J., Janssen F., Wouters A.G.B., Delcour J.A. (2023). Air-Water Interfacial and Foaming Properties of Native Protein in Aqueous Quinoa (*Chenopodium quinoa* Willd.) Extracts: Impact of PH- and Heat-Induced Aggregation. Food Hydrocoll..

[41] Filip D., Macocinschi D., Nica S.L., Condurache B., Stoleru E., Mihaela D., Bargan A., Zaltariov M. (2023). Hybrid green bionanocomposites based on chitosan/starch/gelatin and metallic nanoparticles for biological applications. Int. J. Biol. Macromol..

[42] Kumar P., Verma A.K., Umaraw P., Mehta N., Ismail-Fitry M.R., Narnoliya L.K., Sharma N., Sazili A.Q., Ravishankar G.A., Ranga Rao A., Tahergorabi R., Mohan A.B.T.-H. (2024). Chapter 11—Health Implications of Plant-Based Meat Analogs. Handbook of Plant-Based Meat Analogs: Innovation, Technology and Quality.

[43] Huang K., Liu Y., Zhang Y., Cao H., Luo D., Yi C., Guan X. (2022). Formulation of Plant-Based Yoghurt from Soybean and Quinoa and Evaluation of Physicochemical, Rheological, Sensory and Functional Properties. Food Biosci..

[44] Shi L., Tianqi F., Zhang C., Deng X., Zhou Y., Wang J., Wang L. (2023). High-Protein Compound Yogurt with Quinoa Improved Clinical Features and Metabolism of High-Fat Diet–Induced Nonalcoholic Fatty Liver Disease in Mice. J. Dairy. Sci..

[45] Aguiar E.V., Santos F.G., Centeno A.C.L.S., Capriles V.D. (2021). Influence of Pseudocereals on Gluten-Free Bread Quality: A Study Integrating Dough Rheology, Bread Physical Properties and Acceptability. Food Res. Int..

[46] Burbano J.J., Di Pierro J.P., Camacho C., Vidaurre-Ruiz J., Repo-Carrasco-Valencia R., Iglesias F.A., Sánchez M., Ospina Y.A.M., Igartúa D.E., Correa M.J. (2025). Extruded Quinoa Flour Applied for the Development of Gluten-Free Breads: A Technological, Sensory and Microstructural Approach. Plant Foods Hum. Nutr..

[47] Peñalver R., Nieto G. (2024). Developing a Functional Gluten-Free Sourdough Bread by Incorporating Quinoa, Amaranth, Rice and Spirulina. LWT.

[48] Vidaurre-Ruiz J., Vargas R.J.Y., Alcázar-Alay S., Encina-Zelada C.R., Cabezas D.M., Correa M.J., Repo-Carrasco-Valencia R. (2022). Andean Crops: Kañiwa and Tarwi Flours Used for the Development of Vegan Gluten-Free Muffins. J. Sci. Food Agric..

[49] Villalobos-Fernandez L., Inga M., Betalleluz-Pallardel I. (2025). Properties and Emulsifying Performance of Octenyl Succinic Anhydride-Modified Starch from an Andean Pseudocereal: Cañihua (*Chenopodium pallidicaulle* Aellen). Carbohydr. Polym. Technol. Appl..

[50] Zhang X., Li Z., Zheng X., Wen W., Wang X. (2024). Characteristics of Quinoa Protein Isolate Treated by Pulsed Electric Field. Foods.

[51] De-La-Cruz-Yoshiura S., Vidaurre-Ruiz J., Alcázar-Alay S., Encina-Zelada C.R., Cabezas D.M., Correa M.J., Repo-Carrasco-Valencia R. (2023). Sprouted Andean Grains: An Alternative for the Development of Nutritious and Functional Products. Food Rev. Int..

[52] He Y., Deng Z., Chai T., Yang M., Liu J., Liu H. (2025). Germination Affects Structural and Techno-Functional Properties of Proteins from Quinoa Seeds with Increased Realease of Antioxidant Peptides by Gastrointestinal Digestion. Food Chem..

[53] Filannino P., Di Cagno R., Gobbetti M. (2018). Metabolic and Functional Paths of Lactic Acid Bacteria in Plant Foods: Get out of the Labyrinth. Curr. Opin. Biotechnol..

[54] Chirinos R., Ochoa K., Aguilar-Galvez A., Carpentier S., Pedreschi R., Campos D. (2018). Obtaining of Peptides with in Vitro Antioxidant and Angiotensin I Converting Enzyme Inhibitory Activities from Cañihua Protein (*Chenopodium pallidicaule* Aellen). J. Cereal Sci..

[55] Cruz-Casas D.E., Aguilar C.N., Ascacio-Valdés J.A., Rodríguez-Herrera R., Chávez-González M.L., Flores-Gallegos A.C. (2023). Bioactive Protein Hydrolysates Obtained from Amaranth by Fermentation with Lactic Acid Bacteria and Bacillus Species. Heliyon.

[56] Mohamed I.O. (2023). Interaction of Starch with Some Food Macromolecules during the Extrusion Process and Its Effect on Modulating Physicochemical and Digestible Properties. A Review. Carbohydr. Polym. Technol. Appl..

[57] Wang Q., Li L., Wang T., Zheng X. (2022). A Review of Extrusion-Modified Underutilized Cereal Flour: Chemical Composition, Functionality, and Its Modulation on Starchy Food Quality. Food Chem..

[58] Quispe A.J., Moreno M.C., Leon A.M., Bouchon P., Medina W.T. (2024). Design of Cañihua-Rice: Development and Characterization of an Analogue of Rice by Warm-Extrusion of Cañihua (*Chenopodium pallidicaule* Aellen) and Rice (*Oryza sativa*) Flours. Food Humanit..

[59] Ramos Diaz J.M., Suuronen J.P., Deegan K.C., Serimaa R., Tuorila H., Jouppila K. (2015). Physical and Sensory Characteristics of Corn-Based Extruded Snacks Containing Amaranth, Quinoa and Kañiwa Flour. LWT.

[60] Chao C., Lee J.H., Kim H.W., Kim I.W., Park H.J., Lee S.H. (2025). Effect of Mealworm Component and Extrusion Temperature on Fibrous Structure Formation and Physicochemical Properties of Meat Analog Extrudates. Food Chem..

[61] Kantanen K., Chamlagain B., Succi V., Seppänen S., Edelmann M., Siitonen A., Kemppinen L., Kemppinen A., Kangas M., Varmanen P. (2024). Fermentation of Faba Bean Flour as a Pre-Treatment in High-Moisture Extrusion for Simultaneous Fortification of Vitamin B12 and Reduction of Raffinose Oligosaccharides in Meat Analogues. Future Foods.

[62] Zare L., Mollakhalili-Meybodi N., Fallahzadeh H., Arab M. (2022). Effect of Atmospheric Pressure Cold Plasma (ACP) Treatment on the Technological Characteristics of Quinoa Flour. LWT.

[63] Zarkar S., Kalaivendan R.G.T., Eazhumalai G., Annapure U.S. (2024). Atmospheric Pin-to-Plate Cold Plasma Modification of Amaranth Starch & Its Application as a Stabilizer in Low-Fat Mayonnaise. Int. J. Biol. Macromol..

[64] Mu H., Xue S., Sun Q., Shi J., Zhang D., Wang D., Wei J. (2023). Research Progress of Quinoa Seeds (*Chenopodium quinoa* Wild.): Nutritional Components, Technological Treatment, and Application. Foods.

[65] Hosseini M., Farahmandfar R., Motamedzadegan A., Mollakhalili-meybodi N., Lai W.F. (2025). Modifying the Techno-Functional Characteristics of Quinoa Protein Isolate by Atmospheric Cold Plasma (ACP). Food Hydrocoll..

[66] Sharma A., Kashyap S., Singh S. (2025). Exploring the Advances in Quinoa Processing: A Comprehensive Review Enhancing Nutritional Quality and Health Benefits along with Industrial Feasibility of Quinoa. Food Res. Int..

[67] Condés M.C., Añón M.C., Mauri A.N. (2015). Amaranth Protein Films Prepared with High-Pressure Treated Proteins. J. Food Eng..

[68] Muntean M.-V., Marian O., Barbieru V., Cătunescu G.M., Ranta O., Drocas I., Terhes S. (2016). High Pressure Processing in Food Industry—Characteristics and Applications. Agric. Agric. Sci. Procedia.

[69] Zhu F., Li H. (2019). Effect of High Hydrostatic Pressure on Physicochemical Properties of Quinoa Flour. LWT.

[70] Luo L., Yang Z., Wang H., Ashokkumar M., Hemar Y. (2022). Impacts of Sonication and High Hydrostatic Pressure on the Structural and Physicochemical Properties of Quinoa Protein Isolate Dispersions at Acidic, Neutral and Alkaline PHs. Ultrason. Sonochem..

[71] Thakur D., Bareen M.A., Sahu J.K., Saha S. (2024). Effect of High-Pressure Treatment and Germination on the Functional, Rheological, and Microstructural Characteristics of Red Quinoa (*Chenopodium quinoa*) Gels. Food Hydrocoll..

[72] Ahmed J., Thomas L. (2020). Changes in Structural, Functional and Antioxidant Properties Induced by High Pressure on Quinoa Flour. J. Food Meas. Charact..

[73] Kulikova A.S., Leiberova A.K., Okechukwu Q.N., Ravishankar G.A., Rao A.R., Kovaleva E.G. (2024). Pea as a Key Ingredient in Plant-Based Meat Analogs: A Comprehensive Treatise. Handbook of Plant-Based Meat Analogs.

[74] Mateen A., Singh G. (2023). Evaluating the Potential of Millets as Blend Components with Soy Protein Isolate in a High Moisture Extrusion System for Improved Texture, Structure, and Colour Properties of Meat Analogues. Food Res. Int..

[75] Ozdal T., Abu-Khalil F. (2024). Pseudocereal Protein—Application and Health Benefits.

[76] Luo J., Liu S., Wang Y., Chen Q., Shi Y. (2025). Improvement of Compositional, Textural, and Rheological Characteristics in Plant-Based Cheese Analogs Fermented by Kefir Grain. Food Chem..

[77] Alfieri F., Rivero-Pino F., Zakidou P., Fernandez-Dumont A., Roldán-Torres R. (2023). Processes for Obtaining Plant-Based Dairy and Meat Substitutes. Sustainable Food Science—A Comprehensive Approach.

[78] Suárez S.E., Rabesona H., Ménard O., Jardin J., Anton M., Cristina Añón M. (2024). Dynamic Digestion of a High Protein Beverage Based on Amaranth: Structural Changes and Antihypertensive Activity. Food Res. Int..

[79] Rodriguez J.P., Bonifacio A., Gómez-Pando L.R., Mujica A., Sørensen M. (2023). Cañahua (*Chenopodium pallidicaule* Aellen). Neglected and Underutilized Crops: Future Smart Food.

[80] Córdoba-Cerón D.M., Bravo-Gómez J.E., Agudelo-Laverde L.M., Roa-Acosta D.F., Nieto-Calvache J.E. (2023). Techno-Functional Properties of Gluten-Free Pasta from Hyperprotein Quinoa Flour. Heliyon.

[81] D’Amico S., Schoenlechner R. (2017). Amaranth: Its Unique Nutritional and Health-Promoting Attributes. Gluten-Free Ancient Grains.

[82] Feitosa B.F., Barros J.H.T., Feitoza J.V.F. (2024). Pseudocereals—A Bibliometric Analysis and Literature Review on the Potential for Manufacturing Flours, Bakery Products and Milk Analogues. NFS J..

[83] Kahlon T.S., Avena-Bustillos R.J., Chiu M.C.M. (2016). Sensory Evaluation of Gluten-Free Quinoa Whole Grain Snacks. Heliyon.

[84] Moss R., Barker S., Falkeisen A., Gorman M., Knowles S., McSweeney M.B. (2022). An Investigation into Consumer Perception and Attitudes towards Plant-Based Alternatives to Milk. Food Res. Int..

[85] Smetana S., Ristic D., Pleissner D., Tuomisto H.L., Parniakov O., Heinz V. (2023). Meat Substitutes: Resource Demands and Environmental Footprints. Resour. Conserv. Recycl..

[86] Semba R.D., Ramsing R., Rahman N., Kraemer K., Bloem M.W. (2021). Legumes as a Sustainable Source of Protein in Human Diets. Glob. Food. Secur..

[87] Pirzadah T.B., Malik B. (2020). Pseudocereals as Super Foods of 21st Century: Recent Technological Interventions. J. Agric. Food Res..

[88] Quiroga Ledezma C.C. (2020). Native Food Crops for Present and Future Generations: Their Role in Nutrition and Health. Their Role in Nutrition and Health. Sustainability of the Food System: Sovereignty, Waste, and Nutrients Bioavailability.

[89] Winkel T., Bommel P., Chevarría-Lazo M., Cortes G., Del Castillo C., Gasselin P., Léger F., Nina-Laura J.P., Rambal S., Tichit M. (2016). Panarchy of an Indigenous Agroecosystem in the Globalized Market: The Quinoa Production in the Bolivian Altiplano. Glob. Environ. Change.

[90] Mazumder M.A.R., Panpipat W., Chaijan M., Shetty K., Rawdkuen S. (2023). Role of Plant Protein on the Quality and Structure of Meat Analogs: A New Perspective for Vegetarian Foods. Future Foods.

[91] Galindo-Luján R., Pont L., Sanz-Nebot V., Benavente F. (2023). Protein Profiling and Classification of Commercial Quinoa Grains by MALDI-TOF-MS and Chemometrics. Food Chem..

[92] Melini V., Melini F. (2024). Antioxidant Components and Health Benefits of Pigmented Pseudocereals. Pigmented Grains.

[93] Ishaq A., Irfan S., Sameen A., Khalid N. (2022). Plant-Based Meat Analogs: A Review with Reference to Formulation and Gastrointestinal Fate. Curr. Res. Food Sci..

[94] Guo Z., Deng X., Ping C., Li X., Li D., Wu X., Xiao X., Kong R. (2025). Quinoa: Nutritional and Phytochemical Value, Beneficial Effects, and Future Applications. Appl. Food Res..

[95] Hoogstraaten M.J., Frenken K., Vaskelainen T., Boon W.P.C. (2023). Replacing Meat, an Easy Feat? The Role of Strategic Categorizing in the Rise of Meat Substitutes. Environ. Innov. Soc. Transit..

[96] Song L.M., Yu Y., Du L.D., Ji X.Y., Gao H., Cai Y.Q., Li C.J., Xue P. (2024). Does Saponin in Quinoa Really Embody the Source of Its Bitterness?. Food Chem..

